# Comparative Ultrastructure and Ecological Adaptation of Adhesive Setae in Four Species of Longhorn Beetles (Coleoptera)

**DOI:** 10.3390/insects16111125

**Published:** 2025-11-03

**Authors:** Zheng Liu, Yuan-Yuan Lu, Mei-Ying Lin

**Affiliations:** 1 Hebei Key Laboratory of Animal Diversity, Langfang Normal University, Langfang 065000, China; liuzheng@lfnu.edu.cn; 2 State Key Laboratory of Animal Biodiversity Conservation and Integrated Pest Management, Institute of Zoology, Chinese Academy of Sciences, 1 Beichen West Road, Chaoyang District, Beijing 100101, China; luyuanyuan@ioz.ac.cn; 3 Engineering Research Center for Forest and Grassland Disaster Prevention and Reduction/Ecological Security and Protection Key Laboratory of Sichuan Province, School of Life Sciences (School of Ecological Forestry), Mianyang Normal University, 166 Mianxing West Road, Mianyang 621000, China

**Keywords:** Coleoptera, Cerambycidae, Vesperidae, adhesion, ultrastructure, scanning electron microscope

## Abstract

Longhorn beetles mainly harm trees, flowers, and certain crops. They are closely associated with their host plants and often climb on vertical trunks, branches, and the underside of leaves. Their tarsi have numerous micro-scale adhesive setae on the ventral side. These setae provide sufficient force to enable the beetles to adhere firmly to smooth or slightly micro-structured surfaces. This study observed and analyzed the ultrastructures of the adhesive setae of three species from different subfamilies of Cerambycidae and one species from Vesperidae using a scanning electron microscope. The comparative analysis focused on characterizing the types, morphology, distribution patterns and adhesion mechanisms of the adhesive ultrastructures in four longhorn beetle species. The results showed that the longhorn beetles generally had pseudotetramerous tarsi (except *An. chinensis*, which had tetramerous tarsi), and that tarsomeres I–III were expanded and widened to increase the contact area between the tarsi and the substrate. Furthermore, a large number of micro-scale adhesive setae were present on the ventral surface of these tarsomeres. In total, five types of adhesive setae were found in the four species studied. The three species belonging to the Cerambycidae family exhibited adhesive setae with numerous short setules, while in the species *Mantitheus pekinensis* belonging to Vesperidae, there are two types of adhesive setae without any short setules. The differentiation of the adhesive structures in longhorn beetles is shaped by the combined effects of phylogeny, sexual dimorphism, and host-plant adaptations.

## Simple Summary

Longhorn beetles are known to infest a variety of plants, such as trees, flowers, and crops. These insects frequently move on vertical trunks and on the undersides of leaves, aided by specialized microscopic setae that are adhesive and located on the ventral sides of their tarsi. This study used scanning electron microscopy to examine and compare the attachment structures of three species of Cerambycidae beetles and one Vesperidae beetle species. The results revealed that the first three segments of their tarsi are broadened and covered with different forms of adhesive setae. The variation in these adhesive structures appears to be influenced by phylogenetic, ecological, and gender-related factors.

## 1. Introduction

Many studies have shown that some animals can adhere strongly to smooth surfaces, whether they are artificial or natural. For example, geckos can crawl freely on vertical walls. Ladybugs, flies, and ants can crawl and adhere to smooth glass surfaces that are vertical or even suspended upside down [1,2,3,4]. Some species can even generate adhesion forces exceeding 100 times their own weight [5].

This ability is due to the micro-scaled adhesive structures on the ventral side of their feet. By actively controlling these adhesive ultrastructures, the insects can rapidly attach to or detach from smooth surfaces, enabling free movement. In nature, even seemingly “smooth” surfaces possess microscopic textures. When the ultrafine adhesive structures, which a select few animals are provided, make contact with these surfaces, they deform and penetrate deeply into the gaps using extremely small terminals, maximizing contact area and generating strong adhesion [6].

In order to adapt to smooth or slightly structured environment surfaces in nature, two types of adhesive structures have gradually evolved over a long period of evolutionary time: smooth and hairy [6,7,8]. Smooth adhesive structures use the deformability of materials to increase the actual contact area with smooth or slightly structured surfaces as much as possible, for example, locusts have smooth euplantulae, which are flexible pad-like structures without hairs on the ventral side of the tarsi [6]; ants have a smooth arolium between their claws [4]. On the other hand, the hairy adhesive structures increase the actual contact area and the adhesion force by entering into the gaps with the micro-setae. For example, Autumn found that the adhesive setae of geckos have tree-like structures branching with 100–1000 spatulate tips [3,9,10]. Flies use claws and two hairy pulvilli under the claws for attachment [2,11,12,13,14,15]. Most Coleoptera species, except for the family Dytiscidae, which have dozens of suckers, rely on the hairy adhesive soles of tarsomeres to achieve the adhesive function, i.e., there are large numbers of micrometer-sized adhesive setae on the ventral surface of the tarsi [1,6,16,17,18,19,20,21,22]. The adhesive setae of the leaf beetles (Chrysomelidae) are the most developed in Coleoptera [17,20,21].

Cerambycidae and Vesperidae are families within the superfamily Chrysomeloidea, encompassing a total of seven families: Chrysomelidae, Orsodacnidae, Megalopodidae, Cerambycidae, Disteniidae, Vesperidae, and Oxypeltidae [23,24,25,26]. The superfamily Chrysomeloidea is a large phytophagous group in Coleoptera; the relationship between Chrysomeloidea and their host plants is close. Thus, many species from this superfamily have adhesive structures with diverse types of setae. Among the families, most of studies on the adhesive ultrastructures focus on the family Chrysomelidae [8,17,20,21,27,28,29], while research on other families, such as the Cerambycidae and Vesperidae, is still limited to a few taxonomic and morphological studies [1,30,31].

Herein, we focus on the adhesive setae of the families Cerambycidae and Vesperidae, whose species mainly damage trees, flowers, or some crops. They are major pests in agriculture and forestry, and often climb on the vertical stems, branches, and the undersides of leaves. The claws and adhesive ultrastructures at the ventral side of tarsi are a prerequisite for attachment to their host plants with smooth surfaces. Therefore, investigating the morphology, types, and adhesion mechanisms of these ultrastructures is essential for understanding the adaptive evolution of longhorn beetles’ adhesive systems. Furthermore, this research could provide an innovative pest control approach, such as the development of biomimetic anti-adhesion materials that can physically prevent insect attachment. This study aims to link the diversity of adhesive structures to their environmental adaptation and identify subtle functional differences, thereby establishing a solid theoretical foundation for designing novel bio-inspired adhesive devices.

## 2. Materials and Methods

### 2.1. Materials

In this study, scanning electron microscopy was used to observe and analyze the adhesive setae on the tarsi of three species from different subfamilies of the family Cerambycidae and one species from the family Vesperidae. Three replicates were completed for each sex of every species. The specimen information for the first set is provided below (Table 1), while details of the two additional sets of replicates are listed in Appendix A (Table A1).

*Aromia bungii* (Cerambycidae: Cerambycinae), one female specimen was collected in the Beijing Botanical Garden in 2020, one male specimen was collected in Taizhou City, Jiangsu Province in 2022; *Anoplophora chinensis* (Cerambycidae: Lamiinae), one female and one male specimens were collected in Daming Mountain, Guangxi in 2020; *Aegosoma sinicum* (Cerambycidae: Prioninae), one female and one male specimens were collected in Lianhuachi Park in Beijing in 2020; *Mantitheus pekinensis* (Vesperidae), one female specimen was collected in the Beijing Botanical Garden in 2022, and one male specimen was collected in Wangzhuang Village, Shicheng Town, Miyun District, Beijing in 2020.

### 2.2. Terminology

The morphological terminology mostly followed that of Stork [1], Beutel and Gorb [6], and Betz [16].

### 2.3. Scanning Electron Microscopy (SEM)

The protarsi were removed from the body with a blade (the protarsi are the most representative structures during attachment and possess the most complete variety of adhesive setae), cleaned with 2% phosphate-buffered saline, stepwise dehydrated through a graded ethanol series (the ethanol concentrations used were 75%, 85%, 95%, and three times in 100%, respectively), CO_2_ critical point dried, coated with platinum, and then examined and photographed with a HITACHI SU8010 field emission scanning electron microscope (HITACHI Co., Ltd., Tokyo, Japan). The unclean background of the SEM images was post-processed with Adobe Photoshop (Adobe Inc., San Jose, CA, USA).

### 2.4. Morphometry of the Attachment System

The length and width of the setae were measured from the SEM images using Image-J 1.53 (National Institutes of Health, Bethesda, MD, USA). To quantify the density of the setae on the tarsi, a 50 × 50 μm frame was applied on different areas of the SEM images using Image-J 1.53 (National Institutes of Health, Bethesda, MD, USA). All the setae within the frame (50 × 50 μm = 2500 μm^2^) were counted and the calculated mean value (2500 divided by the number of setae) represented the average area a single seta occupied. Each data was measured three times and averaged (n = 3).

## 3. Results

**Figure 1:**
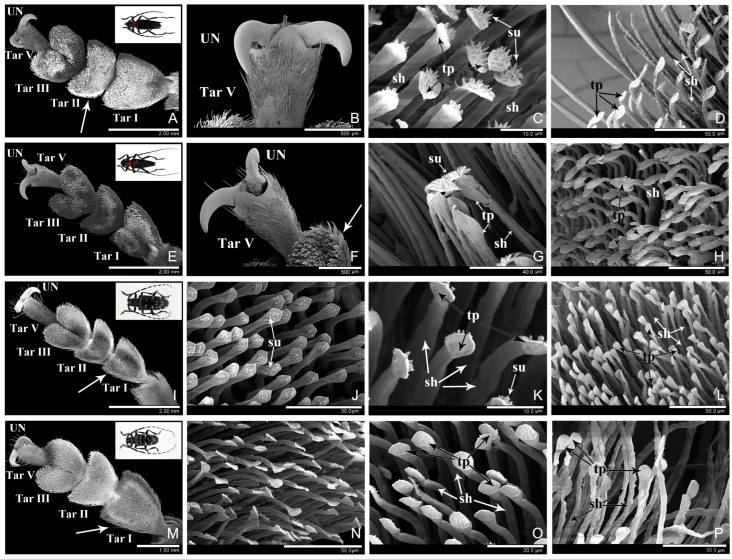
The tarsi and adhesive setae of *Aromia bungii* (**A**–**H**) and *Anoplophora chinensis* (**I**–**P**). (**A**–**D**) female *Ar. bungii*. (**A**) protarsus, ventral side; (**B**) unguis (claws); (**C**) long spindle-shaped setae; (**D**) spatulate setae; (**E**–**H**) male *Ar. bungii*. (**E**) protarsus, ventral side; (**F**) unguis (claws); (**G**) long spindle-shaped setae, ventral side; (**H**) long spindle-shaped setae, dorsal side. (**I**–**L**) female *An. chinensis*. (**I**) protarsus, ventral side; (**J**) spindle-shaped adhesive setae, dorsal side; (**K**) spindle-shaped adhesive setae, ventral side; (**L**) spatulate setae. (**M**–**P**) male *An. chinensis*. (**M**) protarsus, ventral side; (**N**) discoidal seta, dorsal side; (**O**) discoidal seta, ventral side; (**P**) spatulate setae. Abbreviations: UN, unguis (claw); Tar I, the 1st tarsomere; Tar II, the 2nd tarsomere; Tar III, the 3rd tarsomere; Tar V, the 5th tarsomere; sh, setal shaft; tp, terminal plate; su, setule; arrows, non-adhesive setae.

**Figure 2:**
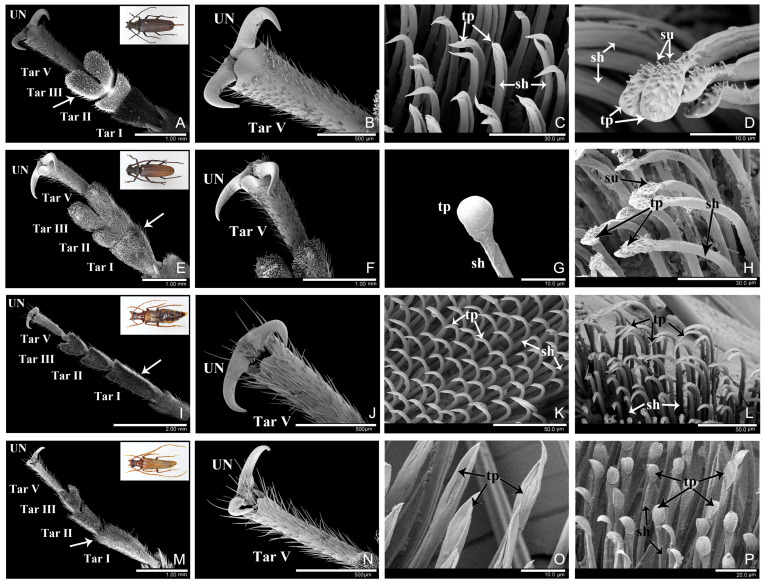
The tarsi and adhesive setae of *Aegosoma sinicum* (**A**–**H**) and *Mantitheus pekinensis* (**I**–**P**). (**A**–**D**) female *Ae. sinicum*. (**A**) protarsus, ventral side; (**B**) unguis (claws); (**C**) tapered setae; (**D**) elliptical setae. (**E**–**H**) male *Ae. sinicum*. (**E**) protarsus, ventral side; (**F**) unguis (claws); (**G**) discoidal setae; (**H**) elliptical setae. (**I**–**L**) female *M. pekinensis*. (**I**) protarsus, ventral side; (**J**) unguis (claws); (**K**,**L**) tapered setae. (**M**–**P**) male *M. pekinensis*. (**M**) protarsus, ventral side; (**N**) unguis (claws); (**O**) tapered setae, ventral side; (**P**) discoidal seta, ventral side. Abbreviations: UN, unguis (claw); Tar I, the 1st tarsomere; Tar II, the 2nd tarsomere; Tar III, the 3rd tarsomere; Tar V, the 5th tarsomere; sh, setal shaft; tp, terminal plate; su, setule; arrows, non-adhesive setae.

**Figure A1:**
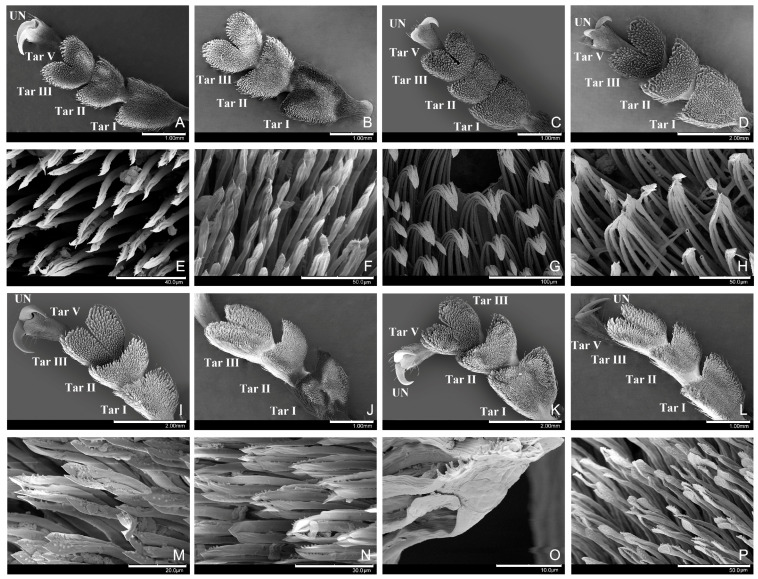
Replicates of the tarsi and adhesive setae of *Aromia bungii* (**A–H**) and *Anoplophora chinensis* (**I–P**). (**A**–**D**) protarsi of *Ar. bungii,* ventral side. (**A**,**B**) female protarsi; (**C**,**D**) male protarsi; (**E**,**F**) long spindle-shaped setae in females, ventral side; (**G**,**H**) long spindle-shaped setae in males, ventral side. (**I**–**L**) protarsi of *An. chinensis,* ventral side. (**I**,**J**) female protarsi; (**K**,**L**) male protarsi; (**M**) spindle-shaped adhesive setae in females, dorsal side; (**N**) Spindle-shaped adhesive setae in females, ventral side; (**O**,**P**) discoidal seta in males, ventral side. Abbreviations: UN, unguis (claw); Tar I, the 1st tarsomere; Tar II, the 2nd tarsomere; Tar III, the 3rd tarsomere; Tar V, the 5th tarsomere.

**Figure A2:**
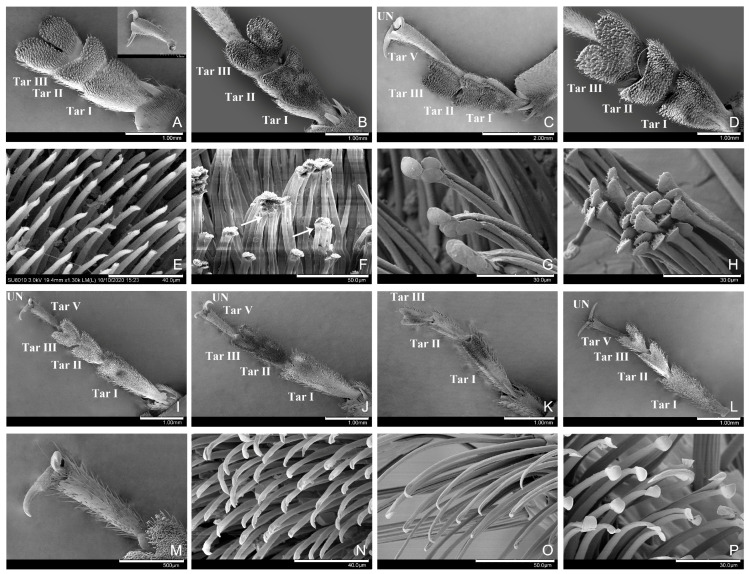
Replicates of the tarsi and adhesive setae of *Aegosoma sinicum* (**A**–**H**) and *Mantitheus pekinensis* (**I**–**P**). (**A**–**D**) protarsi of *Ae. sinicum,* ventral side. (**A**,**B**) female protarsi; (**C**,**D**) male protarsi; (**E**) tapered setae in females; (**F**) elliptical setae in females, ventral side; (**G**) discoidal setae in males, ventral side; (**H**) elliptical setae on the margin of the tarsi in males, ventral side. (**I**–**L**) protarsi of *M. pekinensis,* ventral side. (**I**,**J**) female protarsi; (**K**,**L**) male protarsi; (**M**) unguis (claws) in female; (**N**) tapered setae in females; (**O**) tapered setae in males; (**P**) discoidal setae in males, ventral side. Abbreviations: UN, unguis (claw); Tar I, the 1st tarsomere; Tar II, the 2nd tarsomere; Tar III, the 3rd tarsomere; Tar V, the 5th tarsomere.

In the four observed longhorn beetle species, there were well-developed adhesive structures on the ventral surface of the tarsi. Each tarsus had five tarsomeres with tarsomere IV inconspicuous ventrally, although *An. chinensis* was tetramerous with the tarsomere IV and V completely fused (Figure 1A,E,I,M, Figure 2A,E,I,M and Figure A1A–D,I–L). Tarsomeres I–III were enlarged and widened, and the ventral surface was densely covered with a large number of micro-scaled adhesive setae, forming a hairy adhesive sole (Figure 1A,E,I,M, Figure 2A,E,I,M, and Figure A1A–D,I–L). Tarsomere V was narrow and elongated, with a pair of claws at the end (Figure 1B,F,N, Figure 2B,F,J,N, and Figure A2M).

Adhesive setae differed in size and shape from ordinary non-adhesive setae. The adhesive setae were on the micron-scale, each consisted of a slender setal shaft (sh) and a wide apex (terminal plate, tp) (Figure 1C,D,G,H,J–L,N–P, Figure 2C,D,G,H,K,L,O,P, Figure A1E–H,M–P and Figure A2E–H,M–P). Based on the shape of the apex, setae were categorized into distinct types. In contrast, ordinary non-adhesive setae were larger, featuring sharp tips and no adhesive function. Their tips did not exhibit any expansion (Figure 1A,F,I,M and Figure 2A,E,I,M, arrows).

### 3.1. Aromia bungii (Faldermann, 1835) (Cerambycidae: Cerambycinae) [32]

*Ar. bungii* belongs to the subfamily Cerambycinae. The observed specimens were three females and three males. Tarsomeres I-III (Figure 1A,E and Figure A1A–D) were broad and the area was 1.90, 1.28, and 1.66 mm^2^, respectively. Tarsomere IV was extremely small and not visible from the ventral surface. Tarsomere V was narrow, with long and thick setae. The tarsus had a pair of thick monodentate claws (Figure 1B,F).

The ventral surface of the proximal three tarsomeres was covered with dense adhesive setae (Figure 1A,E and Figure A1A–D). There were two types of setae:

Long spindle-shaped seta: This type of seta was widely distributed on tarsomeres I-III in both females and males and exhibited a density of 1/147.64 μm^2^. The apex of the seta was spindle-shaped (Figure 1C,G,H and Figure A1E–H), with two narrower ends and a wider middle part, measured 22.76 ± 6.85 μm in length and 7.01 ± 0.22 μm in width. More than 20 short setules were present on the dorsal surface of the apex, while the ventral surface was smooth.

Spatulate seta: This type of seta was observed at the distal edge of tarsomere III in females, and exhibited a density of 1/208.33 μm^2^. The apex of the seta was spatula-like shaped or blunt and rounded at the end (Figure 1D), measured 12.15 ± 0.51 μm in length and 6.96 ± 0.58 μm in width. Many short setules were present on the dorsal surface of the apex, while the ventral surface was smooth.

The setae on the lateral surface of the tarsi were fibrous and possessed pointed tips, without adhesive function (Figure 1A,F, arrows).

### 3.2. Anoplophora chinensis (Forster, 1771) (Cerambycidae: Lamiinae) [33]

*A. chinensis* belongs to the subfamily Lamiinae. The observed specimens were three females and three males. The tarsi were tetramerous with tarsomeres IV and V fused. Tarsomeres I–III (Figure 1I,M and Figure A1I–L) were broad and the area was 1.22, 0.85, and 1.03 mm^2^ respectively. Tarsomere V was narrow, with long and thick setae. The tarsus had a pair of thick monodentate claws (Figure 1I,M).

The ventral surface of the proximal three tarsomeres was covered with dense adhesive setae (Figure 1I,M and Figure A1I–L). There were three types of setae:

Spindle-shaped seta: This type of seta was widely distributed on tarsomeres I–III in females, and exhibited a density of 1/69.23 μm^2^. The apex of the seta was spindle-shaped (Figure 1J,K and Figure A1M,N), with two narrower ends and a wider middle part, measured 12.61 ± 3.06 μm in length and 5.26 ± 0.42 μm in width of the apex. More than ten short setules were present on the dorsal surface of the apex, while the ventral surface was V-shaped at the joint with the setal shaft (sh) (Figure 1K and Figure A1N).

Spatulate seta: This type of seta was observed at the distal edge of the tarsomere III in both females and males, and exhibited a density of 1/138.89 μm^2^. The apex of the seta was spatula-like shaped with the distal part wider than the base (Figure 1L,P), measured 11.95 ± 0.64 μm in length and 6.07 ± 0.28 μm in width. Short setules were present on the dorsal surface of the apex, while the ventral surface was smooth and without obvious boundary at the joint with the setal shaft.

Discoidal seta: This type of seta was widely distributed on tarsomeres I–III in males, and exhibited a density of 1/178.67 μm^2^. The apex of the seta was round and disc-shaped (Figure 1N,O and Figure A1O,P), with a diameter of 7.80 ± 0.47 μm (n = 3). More than ten short setules were present on the dorsal surface, while the ventral edge of the disc-apex had a slightly raised border.

The setae on the lateral surface of the tarsi were fibrous and possessed pointed tips, without adhesive function (Figure 1I,M, arrows).

In *An. chinensis*, although the male discoidal setae (Figure 1N,O and Figure A1O,P) appeared similarly to the female spindle-shaped setae (Figure 1J,K) from the dorsal view, they differed in terms of ventral morphology and size. Specifically, the discoidal seta exhibited a pronounced raised border at the junction of its apical ventral surface and the setal shaft (Figure 1O and Figure A1O,P). In contrast, the female spindle-shaped seta formed a “V”-shaped junction at the same position (Figure 1K and Figure A1N). Additionally, the two setae varied in diameter. Meanwhile, the spatulate seta had a smooth, borderless surface (Figure 1L,P).

### 3.3. Aegosoma sinicum White, 1853 (Cerambycidae: Prioninae) [34]

*Aegosoma sinicum* belongs to the subfamily Prioninae. The observed specimens were three females and three males. Tarsomeres I–III were broad and the area was 0.43, 0.37, and 0.64 mm^2^ respectively. Tarsomere IV was extremely small and not visible from the ventral surface. Tarsomere V was narrow, with long and thick setae. The tarsus had a pair of thick monodentate claws (Figure 2B,F).

The ventral surface of the proximal three tarsomeres was covered with dense adhesive setae (Figure 2A,E and Figure A2A–D). There were three types of setae:

Tapered seta: This type of seta was distributed on tarsomere I and II in females, and exhibited a density of 1/145.45 μm^2^. The seta had a straight setal shaft at base, tapered upwards into a curved, acute apex (Figure 2C and Figure A2E), which measured 10.51 ± 2.07 μm in length and 2.76 ± 0.56 μm in width. The dorsal surface of the apex possessed few or no short setules, while the ventral surface was smooth. The tapered setae on tarsomere II were slightly wider than those on tarsomere I, representing an intermediate morphology between the setae on tarsomeres I and III.

Elliptical seta: This type of seta was distributed on tarsomeres III in females and on the margins in males, exhibited a density of 1/156.25 μm^2^. The apex of the seta was elliptical in shape (Figure 2D,H and Figure A2F,H), although slight morphological variation was observed in tips of setae located at different positions, measured 8.60 ± 1.67 μm (n = 3) in length and 6.61 ± 0.84 μm (n = 3) in width. On the dorsal surface of the apex, 15–20 short setules were present. The ventral side had a smooth or “V”-shaped junction between its apex and the setal shaft (Figure A2F,H).

Discoidal seta: This type of seta was distributed in the middle area of tarsomeres I–III in males, and exhibited a density of 1/178.57 μm^2^. The apex of the seta was disc-shaped (Figure 2G and Figure A2G), with a diameter of 8.26 ± 0.62 μm (n = 3). Short setules were absent from the dorsal surface of the discoidal apex, while the ventral surface was concave in the center with a slightly raised arcuate border at the edge.

The setae on the lateral surface of the tarsi were fibrous and possessed pointed tips, without adhesive function (Figure 2A,E, arrows).

### 3.4. Mantitheus pekinensis Fairmaire, 1889 (Vesperidae: Philinae) [35]

*M. pekinensis* belongs to the family Vesperidae and subfamily Philinae. The observed specimens were three females and three males. Compared to the other three species in family Cerambycidae, the tarsi of *M. pekinensis* were relatively narrow and elongated (Figure 2I,M and Figure A2I–L). Tarsomeres I–III were slightly widened and the area was 0.28, 0.14, and 0.14 mm^2^ respectively. Tarsomere IV was minute, and tarsomere V was narrow, with long and thick setae. The tarsus had a pair of sharp monodentate claws (Figure 2J,N and Figure A2M).

The ventral surface of the proximal three tarsomeres was covered with dense adhesive setae (Figure 2I,M and Figure A2I–L). There were two types of setae in males and only one type of setae in females:

Tapered seta: This type of seta was distributed on tarsomeres I–III in females, and on the margin of tarsomere I and the entire ventral surface of tarsomeres II–III in males, exhibited a density of 1/192.31 μm^2^. The setal apex was tapered, gradually becoming pointed and thin at the tip (Figure 2K,L,O and Figure A2N,O), measured 15.73 ± 1.53 μm (n = 3) in length and 3.65 ± 0.51 μm (n = 3) in width. Short setules were absent from the apex.

Discoidal seta: This type of seta was distributed in the center of tarsomere I in males, exhibited a density of 1/208.33 μm^2^. The apex of the seta was oval (Figure 2P and Figure A2P), with the long diameter of 8.58 ± 1.05 μm (n = 3), and the short diameter of 7.25 ± 0.31 μm (n = 3). Short setules were absent from the dorsal surface of the apex, while the ventral surface was concave in the center with a slightly raised edge.

The setae on the lateral surface of the tarsi were fibrous and possessed pointed tips, without adhesive function (Figure 2I,M, arrows).

### 3.5. Comparison of the Adhesive Setae in Four Longhorn Beetle Species

Measurements and statistics were conducted on the terminal area and density of adhesive setae in four longhorn beetle species (Table 2 and Table 3). Each species possessed two or three types of adhesive setae. *Ar. bungii* (subfamily Cerambycinae) had spindle-shaped and spatulate setae, both bearing short setules on the dorsum and occurring at relatively high density. *An. chinensis* (subfamily Lamiinae) had three types of adhesive setae: spindle-shaped, spatulate, and discoidal setae. The discoidal setae were found exclusively in males. *Ae. sinicum* (subfamily Prioninae) had tapered, elliptical, and discoidal adhesive setae. Among them, tapered setae occurred only in females, while discoidal setae occurred only in males. *M. pekinensis* (family Vesperidae) had two types of adhesive setae: tapered and discoidal setae. The discoidal setae were present only in males and featured a smooth dorsal surface without short setules.

Among all types of adhesive setae within each species, the discoidal setae were generally the sparsest and featured a larger terminal area (except for *An. chinensis*, whose spatulate setae at the edge showed a larger area). The sparsest adhesive setae were the discoidal setae in *M. pekinensis* (1/208.33 μm^2^). In contrast, the densest adhesive setae were the spindle-shaped setae in *An. chinensis* (1/69.23 μm^2^), which also had a relatively small terminal area.

Many short setules were present on the dorsal surface of the spindle-shaped seta. This morphological feature may be associated with their high density, as the setules are hypothesized to function as “spacing” devices, preventing adhesion among densely packed setal terminals [1]. Although the spindle-shaped setae of *Ar. bungii* did not exhibit high density, their terminal area was sufficiently large to facilitate adhesion.

### 3.6. Microstructure of the Female Elytral Surface and the Male Behavior During Mating

**Figure 3:**
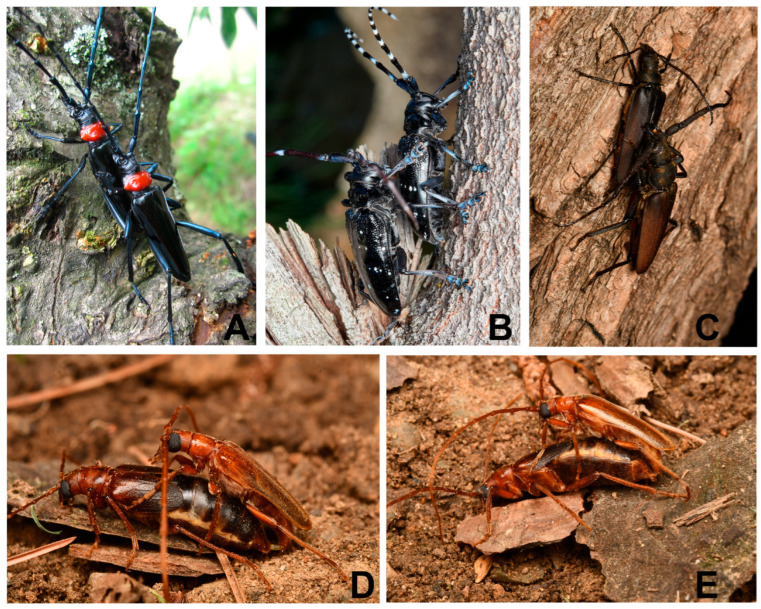
Male behavior during mating. (**A**) *Ar. bungii* on the peach tree (Huanggang, Hubei, China, photographed by Chuangyu Lao). (**B**) *An. chinensis* (Naji Islands, Zhejiang, China, photographed by Suyan Cao). (**C**) *Ae. sinicum* on the willow tree (China National Botanical Garden, Beijing, China, photographed by Dakang Zhou). (**D**,**E**) *M. pekinensis*. (China National Botanical Garden, Beijing, China, photographed by Dakang Zhou).

During mating, longhorn beetles displayed vertical mating postures. Males of the four longhorn beetle species studied pressed the ventral surfaces of their protarsi firmly against the lateral-dorsal areas of the female’s elytra, while simultaneously gripping the elytral margins with their claws (interlocking) (Figure 3). The male *M. pekinensis* attached to the female’s ventral metasternum using its meso-legs, the other three cerambycid species put their meso- and meta-legs on the bark of host plants. Consequently, ultramicroscopic adhesive setae of the protarsi established direct interfacial contact with the female elytra.

**Figure 4:**
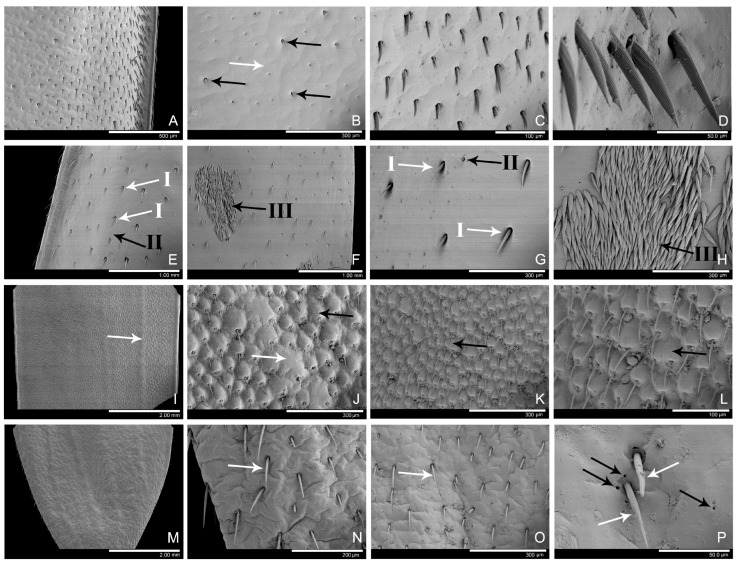
Ultrastructure of the female elytra in four longhorn beetle species. (**A**–**D**) The female elytra of *Ar. bungii*. (**A**) The lateral margins. (**B**) The central area. White arrows, micro-scale irregular structures (**C**,**D**) Setae on the lateral margins. (**E**–**H**) The female elytra of *An. chinensis*. (**E**) The lateral margins. White arrows, the setae of type I. Black arrows, the setae of type II. (**F**) The central area. Black arrows, the setae of type III. (**G**) Setae on the lateral margins. White arrows, the setae of type I. Black arrows, the setae of type II. (**H**) Setal clusters of the white spots. Black arrows, the setae of type III. (**I**–**L**) The female elytra of *Ae. sinicum*. White arrows, the raised ridges. Black arrows, the elliptical sockets. (**I**) The upper-median section of the elytra. (**J**). The longitudinal carina of the elytra. (**K**,**L**). The elliptical seta sockets on the lateral margins. (**M**–**P**). The female elytra of *M. pekinensis*. White arrows, the setae. Black arrows, the pores. (**M**) The median section of the elytra. (**N**) The lateral margins. (**O**,**P**) Setae on the lateral margins.

Structural features on the elytral surface might critically influence the adhesive function. By observing the cuticle surface of female elytra using SEM, it was found that in *Ar. bungii* (Figure 4A–D), robust setae (Figure 4C,D) were densely distributed along the elytral margins where they appeared relatively elongated, while those in the central region (Figure 4B, black arrows) were sparser and markedly shorter. The length of the setae along the elytral margins were from 36.38 to 98.86 μm (n = 3), while those in the central region were 18.63 ± 2.09 μm (n = 3). Micro-scale irregular structures (Figure 4B, white arrows) were observed on the female elytral surface, measuring 105.54 ± 10.69 μm (n = 3) in width.

In *An. chinensis*, the female elytral surface was predominantly smooth (Figure 4E–H), but also bore sparsely distributed setae interspersed with localized dense setal clusters (Figure 4F,H, black arrows). Furthermore, the white spots on the elytra coincided with the distribution of setal clusters, confirming that these markings were produced by the underlying setal structures. Three distinct types of setae were observed: Type I (Figure 4E,G, white arrows) was relatively thick and long, measured from 71.14 to 175.04 μm (n = 3) in length, from 11.38 to 16.15 (n = 3) in width. Type II (Figure 4E,G, black arrows) was considerably thinner and shorter, measured from 55.18 to 80.45 μm (n = 3) in length, from 4.44 to 6.89 μm (n = 3) in width. Type III (forming the setal clusters, Figure 4F,H, black arrows) consisted of densely packed robust setae, measured 104.85 ± 5.79 μm (n = 3) in length and 18.39 ± 2.87 μm (n = 3) in width.

In *Ae. sinicum* (Figure 4I–L), the elytra possessed elliptical sockets (Figure 4J–L, black arrows), from which elongated setae emerged. These seta sockets measured 50.60 ± 6.18 μm (n = 3) in length and 37.17 ± 5.85 μm (n = 3) in width, while the associated setae averaged 61.39 ± 1.68 μm (n = 3) in length. The elytra possessed raised ridges consisted of fused seta sockets (Figure 4I,J, white arrows).

In *M. pekinensis* (Figure 4M–P), female elytra were significantly shortened, failing to cover the abdominal segments. These soft and wrinkled elytra exhibited both setae (Figure 4N–P, white arrows) and pores (Figure 4P, black arrows). Those setae measured from 49.66 to 113.09 μm (n = 3) in length.

In this study, the male-specific adhesive setae observed in *An. chinensis*, *Ae. sinicum* and *M. pekinensis* were the discoidal setae. In *Ar. bungii*, both males and females possessed long spindle-shaped setae. During mating, the male obtained a mechanical interlocking grip on the margin of the female’s elytra using its claws, while simultaneously pressed its adhesive setae firmly against the elytral surface. Adhesive force was enhanced when the setal apex was much smaller than the microstructures of the female’s elytra, thereby maximizing contact area and strengthening adhesion. Despite the inter-specific variation in elytral properties (ranging from hard to soft, smooth to micro-structured), all examined surfaces appeared effectively smooth at the micron-scale relative to the size of the adhesive setae.

## 4. Discussion

### 4.1. Diversity of the Adhesive Ultrastructures in Different Subfamilies of Cerambycidae and Vesperidae

Current understanding of tarsal adhesive ultrastructure in longhorn beetles (Cerambycidae) remains limited. Until now, ultrastructure of the adhesive setae belonging to 11 species of longhorn beetles has been studied, including the four species here. These observations suggest potential taxonomic correlations that require further validation. Comparative analysis of available data reveals similar adhesive setae characteristics within certain lineages, such as the Cerambycinae species *Clytus arietis* [1,30]), *Xylotrechus quadripes* [36], *Rhaphuma horsfieldii* [37], and *Ar. bungii* (this study) share long spindle-shaped setae with short setules on the dorsum; In the subfamily Lamiinae, *An. chinensis* (this study), *Monochamus alternatus* [31], and *Pharsalia antennata* [38] exhibit wider spindle-shaped and spatulate terminals than those of Cerambycinae species. Moreover, Prioninae species *Ae. sinicum* (this study) possesses tapered (in females), elliptical, and discoidal (in males) setae. Lepturinae species *Rhagium mordax* and *Grammoptera ruficornis* [1] that possess spindle-shaped setae with several setules on the dorsal surface and discoidal setae (in males) with “H” or “I” shaped ridges on the dorsal surface. In *M. pekinensis* (Coleoptera: Vesperidae) examined in this study, females possess only tapered setae, whereas males exhibit both tapered and discoidal setae. Based on the limited species, it seems like the adhesive setae within the same family/subfamily level shows a degree of similarities in the basic types of adhesive setae.

### 4.2. Comparison of Adhesive Ultrastructure Between Longhorn Beetles and Leaf Beetles

Longhorn beetles and leaf beetles, both belonging to the phytophagous superfamily Chrysomeloidea, are closely associated with their host plants and possess well-developed adhesive setae. In this study, we compared the similarities and differences in the adhesive structures among longhorn beetles (Cerambycidae and Vesperidae) and leaf beetles (Chrysomelidae), focusing on tarsal morphology, the types of adhesive setae and the short setules discovered on the dorsum of the adhesive setae (Table 4).

The tarsi of the longhorn beetles (Cerambycidae and Vesperidae) and leaf beetles (Chrysomelidae) exhibit morphological similarities: (1) usually possessing pseudotetramerous tarsi with small tarsomere IV (except *An. chinensis* possessing tetramerous tarsus with tarsomeres IV and V fused), among them, tarsomeres I–III are enlarged and widened; (2) their ventral surfaces are covered with a substantial array of micrometer-scaled adhesive setae. These common traits act primarily to increase the contact area between the tarsus and various substrates, whether smooth or micro-structured. Although longhorn beetles (Cerambycidae and Vesperidae) and leaf beetles (Chrysomelidae) have different body size—Cerambycidae and Vesperidae about 15–20 mm, Chrysomelidae normally 5 mm—their adhesive setae have similar tip size about 7–18 um. These micrometer-scale adhesive setae, which can extend into the gaps, conform to substrate topography, and augment effective contact area [6], facilitates adhesion through van der Waals forces and capillary interactions between the setal tips and the interacting surface [9].

Regarding the differences, sexual dimorphism of tarsal morphology is not pronounced in longhorn beetles compared with leaf beetles, which will be elaborated in the next section on sexual dimorphism.

The types of adhesive setae of longhorn beetles and leaf beetles are differentiated by their terminal morphological characteristics. In longhorn beetles, five types of adhesive setae are found, namely spindle-shaped (most common), elliptical, tapered, spatulate and discoidal setae. In contrast, Chrysomelidae predominantly exhibit three distinct types of setae: tapered, spatulate, and discoidal setae (male-specific). Occasionally, branched adhesive setae are observed in specialized lineages such as tortoise beetles [1,21].

In Cerambycid species, many short setules that may function as the spacing devices [1] are on the dorsum of the adhesive setal terminals, contrasting with leaf beetles. *Ar. bungii* (Cerambycidae: Cerambycinae) and *An. chinensis* (Cerambycidae: Lamiinae) exhibited spindle-shaped adhesive setae bearing over 20 short setules on each seta. Furthermore, male *An. chinensis* uniquely possessed discoidal setae featuring more than 10 short setules. This configuration contrasts markedly with leaf beetle morphology, where spatulate adhesive setae bear 1 setule per seta in some Chrysomeline species (such as *Chrysomela populi*) or 10–15 short setules per seta in some Galerucine species (such as *Oides decempunctatus* with more than 10 setules), and discoidal adhesive setae typically bear smooth dorsal surfaces devoid of setular modifications [20,21]. *M. pekinensis* (Vesperidae) exhibits smooth adhesive setae.

### 4.3. Sexual Dimorphism of the Adhesive Setae and Elytral Surface in Four Longhorn Beetles

Both sexes are affected by environmental selective pressures, but males also have to deal with mating-specific functional constraints, especially the need to stick to female elytra. In general, sexual dimorphism of tarsal morphology is less pronounced in longhorn beetles than in leaf beetles. The tarsomere I size is similar for both sexes of longhorn beetles, while the tarsomere I of male leaf beetles is significantly broader than that of conspecific females [17,20,21,39]. Furthermore, all four longhorn beetle species possess simple claws whereas some leaf beetles have accessory claws.

The sexual dimorphism of the adhesive setae exhibits a sequential decrease across these species, in the following order: *M. pekinensis* (most distinct), *Ae. sinicum*, *An. chinensis*, and lastly *Ar. bungii* (least distinct). *M. pekinensis* exhibits more pronounced sexual dimorphism in its adhesive ultrastructures than the other three cerambycid species. This dimorphism is reflected in the tapered setae on female’s tarsomeres I–III, which contrast with the male’s tapered setae and more developed discoidal adhesive setae. This may be related to the greater degree of sexual dimorphism in adults (female with half shortened elytra and micropterous hind wings [40]) and their behavior (females have limited mobility, while the males actively seek out females to mate with). In *Ae. sinicum*, males possess discoidal setae, while females bear tapered setae on tarsomere I. Females’ elliptical setae on tarsomeres III resemble the dorsal aspect of males’ discoidal setae, but differ on the ventral side, where the latter exhibit distinct raised borders. In *An. chinensis*, males possess discoidal and spatulate setae, while females have spindle-shaped and spatulate setal types. The discoidal and spindle-shaped setae exhibit nearly identical dorsal morphology. However, detailed examination of the ventral side reveals that the former has a circular raised border, while the latter forms a distinct V-shaped junction between the tip and the setal shaft. Nevertheless, this sexual dimorphism remains relatively subtle compared with that observed in *Ae. sinicum* and *M. pekinensis*. Adhesive setae of *Ar. bungii* are uniformly spindle-shaped and densely packed, without detectable sexual dimorphism in morphology or arrangement. The longhorn beetles exhibit varying degrees of sex dimorphism—from pronounced to negligible. The underlying causes remain undetermined, though phylogenetic divergence likely represents one contributing factor to this variation.

Longhorn beetles’ discoidal setae, the most representative male-specific “sex setae,” are also prevalent among male leaf beetles [1,17,39], ladybugs [41,42], and even flies, which have a groove under the border facilitating the discharge of secretion [2]. Morphologically analogous structures have also been documented in the arboreal tiger beetle *Neocollyris linearis* [22]. Additionally, males of the ground-dwelling carabid beetles (ground beetles) and cicindelid beetles (non-arboreal tiger beetles) possess other types of “sex setae” [1,22]. Males have evolved specialized sex adhesive setae for strong attachment to the female’s smooth cuticle during copulation, while females lack such structures. These adaptations are hypothesized to enhance mating efficiency by improving grip stability and reducing dislodgment risks [1,43,44,45].

Comparing the contact positions during mating between longhorn beetles (Cerambycidae) and leaf beetles (Chrysomelidae), male longhorn beetles primarily use their protarsi to contact the female’s elytra, whereas the leaf beetles—with shorter legs and compact body shape—utilize both the pro- and meso-tarsi maintaining contact with the dorsal elytral surface, and grasp the elytral edges with claws [17,18]. This behavioral difference may be attributed to the longer legs of cerambycids—their mesotarsi can maintain contact with the substrate (tree trunk) or the female’s metasternum, thereby permitting the protarsi to dedicate their function of elytral contact during copulation.

The examination of the ultrastructure of female elytra and tarsal adhesive contact sites during mating revealed that, although the surface microstructures of elytra in four examined species exhibit significant variation—ranging from sclerotized to relatively soft, smooth (with sparse setae) to surfaces bearing elliptical protrusions or wrinkled textures—the ultramicroscopic adhesive setae demonstrated effective adhesion across all these diverse surfaces. The elytral surfaces could be considered ultramicroscopically smooth because there were no wax deposits (such as the leaf surfaces in *Prunus domestica* and *Chelidonium majus*) or felt-like structures (the leaf surfaces in *Arctium tomentosum*) that would create ultramicro-scale roughness [46,47,48]. The adhesive setae are sufficiently small (measuring several to tens of micrometers in length) to establish effective contact with these surfaces [49,50]. This might indicate that the adhesive setae possess multi-surface adaptability. From an evolutionary perspective, such a multifunctional structure, capable of adapting to diverse complex interfaces rather than being highly specialized, confers greater fitness.

### 4.4. The Relationship Between Adhesive Ultrastructures, Environments and Host Plants

The environment, including host plants, exerts selective pressures on both sexes, manifested through the female’s adhesive setae and the male’s non-sexual setae. In longhorn beetles, both sexes exhibit tarsi densely covered with adhesive setae—a morphological adaptation driven by environmental factors, particularly the smooth cuticular structures of their host plants. This evolutionary trajectory implies that prolonged exposure to plant surfaces has driven the development of enhanced substrate adhesion capabilities [47,51].

Longhorn beetles (Cerambycidae and Vesperidae) and leaf beetles (Chrysomelidae), the two major groups of the superfamily Chrysomeloidea, are highly specialized phytophagous insects that have close ecological relationships with their host plants. They preferentially occupy arboreal and floral surfaces, whose cuticular textures are significantly smoother than those of terrestrial substrates. Thus, all these species possess well-developed adhesive ultrastructures [1], whereas such consistent adaptations contrast sharply with the ecologically distinct (arboreal vs. non-arboreal) families like ground beetles (Carabidae) and tiger beetles (Cicindelidae), whose adhesive structures exhibit disparate environmental adaptation [22].

The relationship between host plants and adhesive setal morphology was examined in the beetle families Vesperidae and Cerambycidae. The Vesperidae species *M. pekinensis* mainly inhabits on gymnosperms, particularly *Pinus bungeana* trunks. Gymnosperms typically have simple, more uniform cuticle structures that facilitate insect attachment, and they often exhibit scaly, exfoliating bark that permits mechanical interlocking with insect claws [52]. In contrast, the examined Cerambycidae species (*Ar. bungii*, *An. chinensis*, and *Ae. sinicum*) predominantly colonize angiosperm hosts: *Ae. sinicum* and *An. chinensis* inhabit on *Salix* and *Populus*, and *Ar. bungii* utilizes diverse hosts including *Prunus* and *Salix*. The cuticles of angiosperms are relatively complex, often featuring wax crystals, felt-like structures, or trichomes (hairs) that serve as defensive mechanisms against insect adhesion [52]. Consequently, these adaptations impose higher demands on insect attachment devices and stronger adhesive forces.

In the morphology of non-sexual adhesive setae, *M. pekinensis* possesses simple tapered setae with only slightly widening terminals, resulting in smaller contact area and weaker adhesion, while the three cerambycid species exhibit more complex setae (long spindle-shaped/spatulate in *Ar. bungii*, spindle-shaped/spatulate in *An. chinensis*, and tapered/elliptical in *Ae. sinicum*) with significantly expanded terminal areas that provide stronger adhesion, indicating a potential link between angiosperm diversification and the evolution of adhesive structures in arboreal beetles.

### 4.5. Bio-Inspired Applications of Biological Adhesion Structures

Biological structures can provide great inspiration for biomimetic design [3,53,54]. For adhesion and locomotion, animals have evolved diverse structural types and functional mechanisms to attach to various surfaces, such as through mechanical interlocking (e.g., claws or spines), with diverse examples in the hairy adhesion structures found in geckos [3] and certain beetles [1,6,7,8,21], the smooth adhesion structures observed in treefrogs [55], and the suckers on octopus tentacles [56]. These biological adhesion systems generally exhibit many advantageous characteristics, such as through mechanical interlocking (e.g., claws or spines), the hairy adhesion structures found in geckos [3] and certain beetles [1,6,7,8,21], the smooth adhesion structures observed in treefrogs [55], and the suckers on octopus tentacles [56]. Their microscopic structures achieve efficient adhesion by maximizing contact area and interfacial interactions (e.g., van der Waals forces and capillary forces), while rapid detachment can be achieved through structural angle adjustment or muscular control [3,57,58,59].

Similarly to geckos, longhorn beetles possess highly specialized “hairy” adhesive setae on their feet that enable them to crawl stably on various complex surfaces. In this study, by examining the morphology of these adhesive setae in detail and analyzing how they contact with various surfaces, such as the surfaces of elytra, host plants, or other environments, we can link the diversity of adhesive structures to their environmental adaptation, and identify subtle functional differences, thereby establishing a solid theoretical foundation for designing novel bio-inspired adhesive devices.

Bio-inspired adhesion technology has significant cross-disciplinary applications and development prospects in various engineering fields, such as biomimetic crawling robots, biomimetic adhesives, bionic medicine, and anti-adhesion materials [56,60,61,62]. In bio-robotics and precision manipulation, bio-inspired adhesive materials are used to develop wall-climbing robots that can adhere to and move on vertical or smooth surfaces. This technology is also applied in Micro-Electro-Mechanical Systems (MEMS) for wafer alignment and micro-manipulation robots to achieve non-destructive grasping and precise positioning at the micro- and nano-scales. In the field of biomedical engineering, it provides novel interface solutions for robotic endoscopes, tissue adhesives, skin patches, and multifunctional bioelectronic devices, significantly enhancing the biocompatibility and operational safety of medical equipment. In sports and safety equipment, this technology can be used to develop anti-slip sports gloves and climbing aids, thereby improving operational stability and personal safety in complex environments through optimized interface contact performance. Our study here enhances the adhesion repository for biodiverse bioinspiration and provides possible combinations for the design of multifunctional adhesive structures.

## 5. Conclusions

Most longhorn beetles exhibit pseudotetramerous tarsi, characterized by small tarsomere IV (except *An. chinensis* that has tetramerous tarsi with tarsomeres IV and V fused). Tarsomeres I–III are progressively expanded, increasing the contact area between the tarsus and the environment surface. The ventral surface of tarsomeres I–III has a large number of micrometer-sized adhesive setae. Five types of adhesive setae were found in this study, namely spindle-shaped, elliptical, spatulate, tapered and discoidal setae. Many adhesive setae of the three cerambycid species observed in this study have a large number of short setules on the dorsal surface of the expanding apex, while *M. pekinensis* in the family Vesperidae has two types of adhesive setae without short setules on the dorsal surface. Comparative evidence implies that phylogenetic divergence, environmental adaptation (host plants), and sexual selection collectively shape the morphological variation in adhesive structures in longhorn beetles. Our findings provide new insights not only into ecological adaptation of longhorn beetles but also into the development of novel adhesive technologies.

## Figures and Tables

**Table 1:** Species of longhorn beetles and their collecting locations.

Family	Subfamily	Species	Collection Site	Collection Time
Cerambycidae	Cerambycinae	*Aromia bungii*	♀ Beijing Botanical Garden, Beijing, China;	♀ 2020;
			♂ Taizhou City, Jiangsu, China	♂ 2022
Cerambycidae	Lamiinae	*Anoplophora chinensis*	♀♂ Daming Mountain, Guangxi, China	♀♂ 2020
Cerambycidae	Prioninae	*Aegosoma sinicum*	♀♂ Lianhuachi Park, Beijing, China	♀♂ 2020
Vesperidae	Philinae	*Mantitheus pekinensis*	♀ Beijing Botanical Garden, Beijing, China;	♀ 2022;
			♂ Miyun District, Beijing, China	♂ 2020

**Table 2:** Setal types and length measurements of four longhorn beetles.

Species	Num. of Setal Types	Discoidal	Spindle-Shaped	Elliptical	Spatulate	Tapered
*Aromia bungii*	2	-	79.76 ± 6.91	-	57.43 ± 7.33	-
*Anoplophora chinensis*	3	39.48 ± 0.82	34.94 ± 4.90	-	55.07 ± 5.83	-
*Aegosoma sinicum*	3	51.85 ± 2.73	-	34.23 ± 3.14	-	24.72 ± 2.68
*Mantitheus pekinensis*	2	61.80 ± 5.54	-	-	-	39.01 ± 4.83

**Table 3:** Density of the adhesive setae in four longhorn beetles (1 seta/* μm^2^).

Species	Num. of Setal Types	Discoidal	Spindle-Shaped	Elliptical	Spatulate	Tapered
*Aromia bungii*	2	-	1/147.64	-	1/192.31	-
*Anoplophora chinensis*	3	1/178.67	1/69.23	-	1/138.89	-
*Aegosoma sinicum*	3	1/178.57	-	1/156.25	-	1/145.45
*Mantitheus pekinensis*	2	1/208.33	-	-	-	1/192.31

* = values representing the average area occupied by a single hair.

**Table 4:** Comparison of adhesive ultrastructure between longhorn beetles and leaf beetles.

Comparison		Longhorn Beetles	Leaf Beetles
Similarities	tarsomeres	pseudotetramerous (except *An. chinensis* tetramerous)	pseudotetramerous
	TarI–III	enlarged	enlarged
	setae	adhesive	adhesive
	setal tip size	7–18 um	7–18 µm
Differences	sexual dimorphism	less pronounced	pronounced
	setal types	spindle-shaped, elliptical, tapered, spatulate, discoidal setae	tapered, spatulate, discoidal setae, branched setae
	short setules	more	less

## Data Availability

The data presented in this study are available on request from the corresponding author.

## Appendix A

**Table A1:** Sampling replicates of longhorn beetle species and their collecting locations.

Family	Subfamily	Species	Collection Site	Collection Time
Cerambycidae	Cerambycinae	*Aromia bungii*	2♀ Baihua Mountain, Beijing, China;	2♀ 2010;
			1♂ Baihua Mountain, Beijing, China;	1♂ 2010;
			1♂ Xiaowutai Mountain, Hebei, China.	1♂ 2023.
Cerambycidae	Lamiinae	*Anoplophora chinensis*	1♀ Qinling National Botanical Garden, Shaanxi, China;	1♀ 2022;
			1♀ Baihua Mountain, Beijing, China;	1♀ 2010;
			1♂ Taibai County, Shaanxi, China;	1♂ 2012;
			1♂ Foping County, Shaanxi, China.	1♂ 2007.
Cerambycidae	Prioninae	*Aegosoma sinicum*	1♀1♂ Linyi, Shandong, China;	1♀1♂ 2025;
			1♀1♂ Baihua Mountain, Beijing, China.	1♀1♂ 2010.
Vesperidae	Philinae	*Mantitheus pekinensis*	2♀ Beijing Botanical Garden, Beijing, China;	2♀ 2011;
			1♂ Miyun District, Beijing, China;	1♂ 2015;
			1♂ Mentougou District, Beijing, China.	1♂ 2018.
